# Peripartum cardiomyopathy: Euro Observational Research Program

**DOI:** 10.1007/s12471-014-0573-5

**Published:** 2014-07-10

**Authors:** M. F. Hoes, I. van Hagen, F. Russo, D. J. Van Veldhuisen, M. P. Van den Berg, J. Roos-Hesselink, K. Y. van Spaendonck-Zwarts, P. van der Meer

**Affiliations:** 1 Department of Cardiology, University Medical Centre Groningen, University of Groningen, Hanzeplein 1, PO Box 30001, 9700 RB Groningen, the Netherlands; 2 Department of Cardiology, Thoraxcenter, Erasmus Medical Centre, PO Box 2040, 3000 CA Rotterdam, the Netherlands; 3 Department of Genetics, Academic Medical Centre Amsterdam, Meibergdreef 9, 1105 AZ Amsterdam, the Netherlands

**Keywords:** Peripartum cardiomyopathy, Heart failure, European peripartum cardiomyopathy registry

## Abstract

Peripartum cardiomyopathy is a rare but potentially life-threatening form of heart failure affecting women late in pregnancy or in the first months after delivery. Peripartum cardiomyopathy is difficult to diagnose and its onset and progression are variable between individuals. The pathophysiology remains poorly understood, hence treatment options are limited and possibly harmful to the foetus. Furthermore, geographical incidence varies greatly and little is known about the incidence in Western countries. To gain further understanding of the pathophysiology and incidence of peripartum cardiomyopathy, the European Society of Cardiology initiated a study group to implement a registry. This review provides an overview of current insights into peripartum cardiomyopathy, highlights the need for such a registry and provides information about this Euro Observational Research Program.

## Introduction

Peripartum cardiomyopathy is a rare but potentially life-threatening form of heart failure affecting women late in pregnancy or in the first months after delivery. Little is known about the prevalence, aetiology and potential therapeutic options. To gain further understanding of the prevalence and pathophysiology of peripartum cardiomyopathy, the European Society of Cardiology (ESC) initiated a study group to implement a registry. This review provides an overview of current insights into peripartum cardiomyopathy, highlights the need for such a registry and provides information about this Euro Observational Research Program.

## Clinical presentation

The incidence of peripartum cardiomyopathy varies geographically, for instance from 1:2300 to 1:4000 in the USA and is as high as 1:300 in Haiti [1, 2]. However, the incidence of peripartum cardiomyopathy in Europe remains unknown. It is characterised by left ventricular dysfunction, manifesting predominantly in the first months postpartum. Peripartum cardiomyopathy is primarily a clinical diagnosis of exclusion. The definition of peripartum cardiomyopathy has changed over the years, but recently the ESC Working Group on peripartum cardiomyopathy has accepted the following definition: ‘an idiopathic cardiomyopathy presenting with heart failure secondary to left ventricular systolic dysfunction towards the end of pregnancy or in the months following delivery, where no other cause of heart failure is found’ [2].

Multiparity, multiple births, older maternal age and prolonged tocolytic therapy are factors found to predispose to the development of peripartum cardiomyopathy. Other suggested risk factors are African origin, family history of cardiomyopathy and pregnancy-induced hypertension [1, 3]. In addition, a recent literature review showed a clear association between the incidence of preeclampsia and peripartum cardiomyopathy [4].

Clinical features consist of dyspnoea on exertion, orthopnoea and peripheral oedema. However, an underlying cause such as ischaemic, valvular or detectable inherited heart disease should not be present. Mild symptoms such as fatigue, palpitations, and abdominal discomfort can be easily misinterpreted as usual postpartum symptoms and associated with sleep deprivation and may delay early diagnosis. Subsequently, patients are often already in New York Heart Association (NYHA) functional class III-IV at first presentation [2].

Electrocardiography (ECG) can show signs of left ventricular hypertrophy with repolarisation abnormalities or other nonspecific signs, but the ECG will hardly ever be normal. In total, 20 % of patients experience non-sustained ventricular arrhythmias [1]. No information is reported on the incidence of sustained ventricular tachycardias. Left ventricular dysfunction is part of the diagnosis, while left ventricular dimensions can be normal in patients with dilated cardiomyopathy. Unfortunately, there is not yet a reliable laboratory test that can confirm the diagnosis. B-type natriuretic peptide can be used, but does not distinguish between different causes of heart failure. Preliminary results revealed microRNA-146a (miR-146a) as a potential biomarker for clinical use, yet to be confirmed in prospective studies [1].

## Pathogenesis of peripartum cardiomyopathy

The underlying molecular mechanisms of peripartum cardiomyopathy remain poorly understood. However, serum markers of apoptosis and inflammation are significantly increased, indicating an impaired adaptive response to the physiological stresses on the heart during pregnancy [3]. Cardiac stress during pregnancy is mainly due to hormones, oxidative stress and volume overload. Regarding the last-mentioned factor, the Janus kinase (JAK)/signal transducers and activators of transcription (STAT) signalling pathway responds almost instantly to stretching neonatal rat and murine cardiomyocytes [5]. STAT3 activation is essential for cardioprotective effects; it leads to induction of the reactive oxygen species (ROS) scavenging enzyme manganese superoxide dismutase (MnSOD) [5].

The role of STAT3 in peripartum cardiomyopathy was discovered in 2007. STAT3 conditional knockout mice developed peripartum cardiomyopathy in a dose-dependent manner (conditional knockout heterozygotes). It was observed that during pregnancy MnSOD levels were increased compared with nulliparous controls, whereas pregnant STAT3 conditional knockout mice showed reduced MnSOD levels. As a consequence, increased ROS levels were found in pregnant STAT conditional knockout mice versus pregnant controls. It was shown that STAT3 can be activated by various signals, including prolactin [6]. Blood levels of the nursing hormone prolactin are substantially increased during pregnancy. Full-length prolactin has proangiogenic effects, whereas the 16 kDa cleaved form elicits antiangiogenic effects. It was determined that cathepsin D is the major prolactin cleaving enzyme in peripartum cardiomyopathy [7]. Furthermore, oxidative stress was found to induce cathepsin D [8]. It is therefore believed that STAT3 deficiency causes a decreased antioxidative activity within cardiomyocytes, leading to increased levels of ROS that in turn activate cathepsin D production. High serum levels of prolactin during pregnancy combined with increased cathepsin D production will therefore result in elevated levels of 16 kDa prolactin leading to cell death and reduced angiogenesis.

Patten et al. provided further evidence for an imbalance between proangiogenic and antiangiogenic factors in peripartum cardiomyopathy. They have also observed that insufficient angiogenesis during pregnancy leads to peripartum cardiomyopathy development [9]. Like STAT3, PGC-1α is a gene that promotes the expression of MnSOD, but unlike STAT3, it also induces the synthesis of vascular endothelial growth factor (VEGF). Thus, in pregnant PGC-1α knockout mice, the 16 kDa prolactin-induced antiangiogenic effect and the inhibition of proangiogenic factors act together towards the development of a very severe form of peripartum cardiomyopathy. They found that PGC-1α knockout mice invariably developed peripartum cardiomyopathy. Deficiency of the major angiogenic mediator PGC-1α in heart leads to decreased secretion of angiogenic factors such as VEGF, thus inhibiting angiogenesis. In late pregnancy, secretion of soluble Flt1 (sFlt1) is increased, which binds and neutralises soluble VEGF [9]. Proangiogenic therapies (i.e. VEGF injections) were found to rescue PGC-1α knockout phenotypes [9]. The observation that women with pre-eclampsia are more frequently affected by peripartum cardiomyopathy supports a role of oxidative stress in the pathogenesis of peripartum cardiomyopathy [9].

**Fig. 1:**
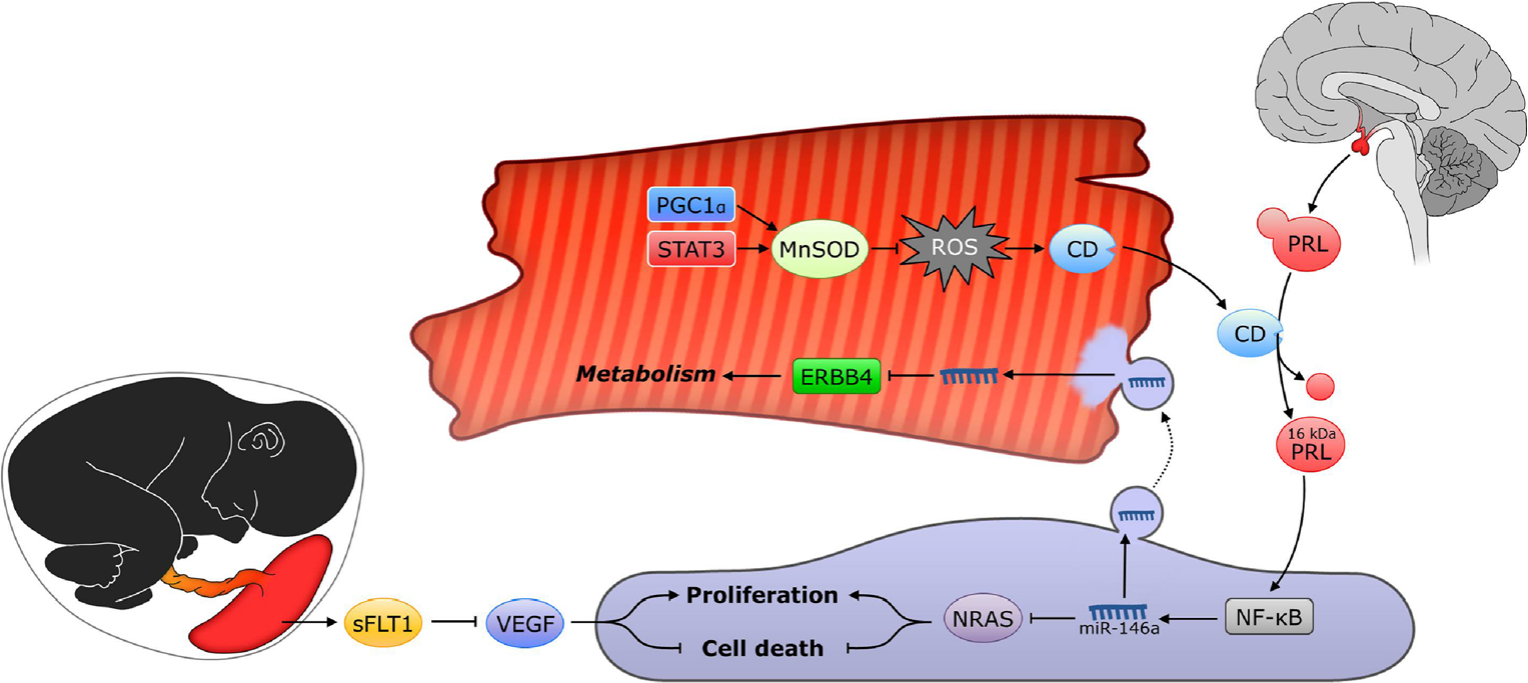
Multiple mechanisms lead to the development of PPCM in mice. Changes in the PGC1α or STAT3 expression lead to aberrant activation of MnSOD and lead to inadequate antioxidant activity in cardiomyocytes (red cell). Increased levels of ROS lead to increased cathepsin D (CD) production. When cathepsin D is secreted into the blood and interacts with prolactin (PRL) that is secreted by the pituitary gland during pregnancy, it cleaves PRL to form 16 kDa prolactin. This truncated form of prolactin can activate the NF-κB pathway in endothelial cells (blue cell) and ultimately induces transcription of microRNA-146a (miR-146a). Endothelial exosomes are loaded with miRNA-146a and, following secretion, are taken up by surrounding cardiomyocytes where it inhibits metabolic activities via *Erbb4, Nras, Notch1, and Irak1*. Additionally, MiRNA-146a inhibits proliferation and promotes cell death in endothelial cells. Combined with inhibition of vascular endothelial growth factor (VEGF) by sFLT1 secretion by the placenta, this subsequently leads to angiogenic imbalance, further deteriorating cardiac function during pregnancy

In line with these findings, Halkein et al. have found miR-146a to be the link between angiogenic imbalance and cardiomyocyte dysfunction [10]. When endothelial cells were incubated with 16 kDa prolactin, miR-146a levels increased, which resulted in reduced angiogenesis. Subsequently exosomal transfer of miR146-a to cardiomyocytes reduced metabolism. Blocking miR-146a with a specific antagomir causes the attenuation of peripartum cardiomyopathy clinical features in STAT3 conditional knockout mice, without affecting full-length prolactin-dependent milk production functions [10]. Furthermore, in patients with peripartum cardiomyopathy miR-146a levels were increased even compared with patients with dilated cardiomyopathy. Taking these results together, it appears that reduced angiogenesis at least partially explains the development of peripartum cardiomyopathy and that miR-146a might be a useful biomarker in the diagnosis. Further work is needed to investigate whether this miRNA can serve as a therapeutic target. Figure 1 provides a summary of the pathophysiological mechanisms underlying peripartum cardiomyopathy in mice, which remain to be confirmed in humans.

## Genetic influences

Classified in 2008 by the ESC as a nonfamilial, nongenetic form of dilated cardiomyopathy associated with pregnancy [11], in recent years a growing body of evidence suggests that part of peripartum cardiomyopathy has a genetic basis. Two Western studies and one South African case series showed the occurrence of peripartum cardiomyopathy cases in familial dilated cardiomyopathy [12]. Accordingly, a positive family history of cardiomyopathy was seen in 16.5 % (19/115) of peripartum cardiomyopathy cases in a German peripartum cardiomyopathy cohort. Recently, more extensive genetic analysis in families with both dilated cardiomyopathy and peripartum cardiomyopathy from various parts of the world revealed a high yield of mutations in cardiomyopathy-related genes, especially in TTN, the gene encoding for titin. Pathogenic mutations were found in 4/18 families (22 %), and mutations that may be pathogenic were found in another 6/18 families (33 %). The majority of identified (likely) pathogenic mutations (7/10) were in the TTN gene. Measurements of passive force in single cardiomyocytes and titin isoform composition supported the pathogenicity of one of the likely pathogenic TTN mutations [13]. The mechanisms underlying the onset of peripartum cardiomyopathy remain unclear, but certainly genetics is part of a complex interplay of different factors. Most probably cardiomyopathy-related genes are not exclusively involved. Moreover, a small genome-wide association study on 41 American peripartum cardiomyopathy samples showed an association with rs258415, in locus 12p11.22 close to the ParaThyroid Hormone-Like Hormone (PTHLH) gene [14]. How these findings might functionally link to peripartum cardiomyopathy is still unclear. However, the geographical variability of peripartum cardiomyopathy incidence suggests that genetics plays an important role in peripartum cardiomyopathy onset.

So far, small patient groups and techniques with limited throughput have been evident restrictions in genetic studies. New genomic techniques, such as next-generation sequencing, now make it possible to analyse larger numbers of genes, even the entire exome or genome, quickly and at reasonable costs. It would be most interesting to perform extensive genetic studies on larger numbers of peripartum cardiomyopathy patients from several parts of the world, to identify (lower penetrant) genetic factors involved in peripartum cardiomyopathy, possibly including factors that underlie geographical variations in incidence and/or clinical characteristics.

## Treatment options

Heart failure in the context of peripartum cardiomyopathy should be treated according to the guidelines. Pharmacological agents such as ACE inhibitors or ARBs are essential, but due to potential teratogenic consequences they can only be given after delivery. Beta-blockers have potential side effects for the neonate as well. Birth weight can be lower and hypoglycaemia and bradycardia postpartum are described. Neonatal monitoring after birth is therefore important. In case of a left ventricular ejection fraction below 35 %, anticoagulants should be considered, also because of the hypercoagulable state of the peripartum patient. A comprehensive therapeutic algorithm for acute heart failure in peripartum cardiomyopathy is given in Fig. 1 in the article by Bachelier-Walenta et al. [1].

A recent randomised controlled trial found that administration of bromocriptine has a beneficial effect on NYHA functional class and left ventricular function in patients with peripartum cardiomyopathy, which however, needs to be confirmed by larger trials as it concerns a relatively small pilot study[15]. Bromocriptine is a dopamine-2D mimetic that inhibits prolactin production. It reduces the stimulating effect of prolactin on processes such as apoptosis, degeneration of the cardiac capillary network and vasoconstriction and has previously been shown to prevent mice from developing peripartum cardiomyopathy.

If a patient is haemodynamically unstable, despite extensive inotropic support, implantation of a left ventricular assist device should be considered, either as a bridge-to-transplant, or as a bridge-to-recovery.

### Delivery

A spontaneous vaginal delivery is possible in a stable patient, with pain management to limit large haemodynamic changes caused by stress. Elective caesarean section carries a slightly increased risk of thromboembolic or haemorrhagic events and is associated with larger amounts of blood loss. In case of haemodynamic instability, the most important treatment for the mother is ending the pregnancy. This also enables optimal medical treatment [1, 3].

### Prognosis

In contrast to other forms of cardiomyopathy, left ventricular dysfunction is reversible in a proportion of the patients with peripartum cardiomyopathy. In 23–54 % of the patients total recovery of ventricular function occurs, more often in patients with a more preserved ejection fraction at first presentation and mainly within 6–12 months. However, recovery can still be achieved beyond 1 year after first presentation [2, 3]. Possible predictors for poor outcome are a dilated left ventricle, low ejection fraction, low body mass index and low cholesterol [1].

The reported overall long-term (47–56 months) mortality varies from 3.3 to 30 % (this tends to be lower in the USA compared with developing countries such as Haiti and South-Africa) [2]. Larger prospective and international studies are needed to be able to accurately estimate mortality and morbidity risks. The recurrence risk of peripartum cardiomyopathy is 30–50 % in subsequent pregnancies [3]. Although not based on solid data, the current consensus is that patients should be advised against a new pregnancy if left ventricular function including diastolic function is still compromised [2]. Left ventricular dysfunction due to peripartum cardiomyopathy is associated with a further decrease of ventricular function and symptoms of heart failure in every following pregnancy [3].

## Peripartum cardiomyopathy registry

To gain further understanding of the prevalence and pathophysiology of peripartum cardiomyopathy, a registry is needed. Several years ago this was already mentioned by Lok et al. who proposed a national survey [16]. At the same time the ESC initiated a working group to carry out a worldwide survey [11]. In this worldwide survey, data are currently being collected on possible risk factors, diagnosis, mode of delivery, standard management and therapeutic interventions currently performed in each centre for patients presenting with signs and symptoms of peripartum cardiomyopathy (www.escardio.org/guidelines-surveys/eorp/surveys/ppcm). The study is hosted, monitored and financed by the ESC and governed by the members of the executive committee. The survey is new and additional to the existing pregnancy registry focusing on women with structural and ischaemic heart disease (ROPAC) (www.escardio.org/guidelines-surveys/eorp/surveys/pregnancy). The Euro Observational Research Program will allow a comparison of women from around the world, presenting with peripartum cardiomyopathy and will report on their 6- and 12-month outcome. In literature, especially data on peripartum cardiomyopathy from Western countries is lacking. Therefore we would like to encourage Dutch cardiologists to enter cases in this registry. New centres volunteering to participate in the study will be accepted; however, cases can also be reported to the authors of this manuscript and they will enter the cases for you. Until now, approximately 180 cases have been entered into the database. The study will continue until 1000 patients are included.

The only inclusion criteria are: diagnosis of peripartum cardiomyopathy, signs and symptoms of heart failure and left ventricular ejection fraction <45 %. Patients can be enrolled retrospectively up to 6 months after diagnosis.

## Conclusions

Peripartum cardiomyopathy can be fatal and occurs at a very emotional time for (future) parents. Peripartum cardiomyopathy is difficult to diagnose since symptoms of peripartum cardiomyopathy are to some extent similar to symptoms of a normal pregnancy. Disease onset and progression are variable between individuals and several factors have been found to increase the risk of peripartum cardiomyopathy development. Moreover, no reliable laboratory tests are currently available to confirm the diagnosis. Treatment options are limited due to possible harmful side effects for the foetus. The exact mechanism underlying peripartum cardiomyopathy remains poorly understood, but a cardiac angiogenic imbalance may play an important role.

As it is difficult to diagnose and geographical incidences vary greatly, it is imperative to gain further understanding of disease progression, recurrence risk, and development of treatment options. We encourage Dutch cardiologists to enter peripartum cardiomyopathy patients into the worldwide pregnancy registry in order to get a clearer view of this condition.

## Funding

Karin Y. van Spaendonck is supported by the ICIN—Netherlands Heart Institute. Peter van der Meer is supported by the Dutch Heart Foundation (grant: 2012 T047), Netherlands Organisation for Scientific Research and the ICIN—Netherlands Heart Institute.

## References

[1] Bachelier-Walenta K, Hilfiker-Kleiner D, Sliwa K (2013). Peripartum cardiomyopathy: update 2012. Curr Opin Crit Care.

[2] Sliwa K, Hilfiker-Kleiner D, Petrie MC (2010). Current state of knowledge on aetiology, diagnosis, management, and therapy of peripartum cardiomyopathy: a position statement from the heart failure association of the european society of cardiology working group on peripartum cardiomyopathy. Eur J Heart Fail.

[3] Sliwa K, Fett J, Elkayam U (2006). Peripartum cardiomyopathy. Lancet.

[4] Bello N, Rendon IS, Arany Z (2013). The relationship between pre-eclampsia and peripartum cardiomyopathy: a systematic review and meta-analysis. J Am Coll Cardiol.

[5] Negoro S, Kunisada K, Fujio Y (2001). Activation of signal transducer and activator of transcription 3 protects cardiomyocytes from hypoxia/reoxygenation-induced oxidative stress through the upregulation of manganese superoxide dismutase. Circulation.

[6] Cataldo L, Chen NY, Yuan Q (2000). Inhibition of oncogene STAT3 phosphorylation by a prolactin antagonist, hPRL-G129R, in T-47D human breast cancer cells. Int J Oncol.

[7] Hilfiker-Kleiner D, Kaminski K, Podewski E (2007). A cathepsin D-cleaved 16 kDa form of prolactin mediates postpartum cardiomyopathy. Cell.

[8] Corbacho AM, Martinez De La Escalera G, Clapp C (2002). Roles of prolactin and related members of the prolactin/growth hormone/placental lactogen family in angiogenesis. J Endocrinol.

[9] Patten IS, Rana S, Shahul S (2012). Cardiac angiogenic imbalance leads to peripartum cardiomyopathy. Nature.

[10] Halkein J, Tabruyn SP, Ricke-Hoch M (2013). MicroRNA-146a is a therapeutic target and biomarker for peripartum cardiomyopathy. J Clin Invest.

[11] Sliwa K, Hilfiker-Kleiner D, Mebazaa A, et al. EuroObservational research program: a worldwide registry on PPCM. Eur J Heart Fail 2014;In Press 10.1002/ejhf.68 24591060

[12] van Spaendonck-Zwarts KY, van Tintelen JP, van Veldhuisen DJ (2010). Peripartum cardiomyopathy as a part of familial dilated cardiomyopathy. Circulation.

[13] van Spaendonck-Zwarts KY, Posafalvi A, van den Berg MP, et al. Titin gene mutations are common in families with both peripartum cardiomyopathy and dilated cardiomyopathy. Eur Heart J 2014 [Epub ahead of print] 10.1093/eurheartj/ehu050 24558114

[14] Horne BD, Rasmusson KD, Alharethi R (2011). Genome-wide significance and replication of the chromosome 12p11.22 locus near the PTHLH gene for peripartum cardiomyopathy. Circ Cardiovasc Genet.

[15] Sliwa K, Blauwet L, Tibazarwa K (2010). Evaluation of bromocriptine in the treatment of acute severe peripartum cardiomyopathy: a proof-of-concept pilot study. Circulation.

[16] Lok SI, Kirkels JH, Klöpping C (2011). Peripartum cardiomyopathy: the need for a national database. Neth Heart J.

